# Association between sleep difficulties as well as duration and hypertension: is BMI a mediator?

**DOI:** 10.1017/gheg.2017.10

**Published:** 2017-08-29

**Authors:** R. M. Carrillo-Larco, A. Bernabe-Ortiz, K. A. Sacksteder, F. Diez-Canseco, M. K. Cárdenas, R. H. Gilman, J. J. Miranda

**Affiliations:** 1 CRONICAS Center of Excellence in Chronic Diseases, Universidad Peruana Cayetano Heredia, Lima, Peru; 2 School of Public Health and Administration, Universidad Peruana Cayetano Heredia, Lima, Peru; 3 Department of International Health, Bloomberg School of Public Health, Johns Hopkins University, Baltimore, USA; 4 Área de Investigación y Desarrollo, Asociación Benéfica PRISMA, Lima, Peru; 5 Department of Medicine, School of Medicine, Universidad Peruana Cayetano Heredia, Lima, Peru

**Keywords:** Body mass index, hypertension, obesity, sleep apnea, sleep disorders

## Abstract

Sleep difficulties and short sleep duration have been associated with hypertension. Though body mass index (BMI) may be a mediator variable, the mediation effect has not been defined. We aimed to assess the association between sleep duration and sleep difficulties with hypertension, to determine if BMI is a mediator variable, and to quantify the mediation effect. We conducted a mediation analysis and calculated prevalence ratios with 95% confidence intervals. The exposure variables were sleep duration and sleep difficulties, and the outcome was hypertension. Sleep difficulties were statistically significantly associated with a 43% higher prevalence of hypertension in multivariable analyses; results were not statistically significant for sleep duration. In these analyses, and in sex-specific subgroup analyses, we found no strong evidence that BMI mediated the association between sleep indices and risk of hypertension. Our findings suggest that BMI does not appear to mediate the association between sleep patterns and hypertension. These results highlight the need to further study the mechanisms underlying the relationship between sleep patterns and cardiovascular risk factors.

## Introduction

To lessen the global burden of cardiovascular diseases, it is crucial that rates of hypertension and other cardiovascular risk factors are reduced [1, 2]. Studying the biological mechanisms underlying the risk factors for hypertension is needed to design more effective interventions to manage hypertension. Important risk factors for hypertension include short sleep duration and sleep difficulties [3–5]. Experimental studies have shown that acute sleep restriction is associated with higher blood pressure during the night and morning, as well as associated with higher sensitivity of arterial baroreflex responses [6]. Moreover, randomized controlled trials have shown a reduction in blood pressure as well as in cardiovascular events in patients with obstructive sleep apnea treated with continuous positive airway pressure [7, 8]. Nevertheless, these studies did not include weight management as a key treatment to improve the effect of sleep health on blood pressure. It has been suggested that body mass index (BMI) and obesity are partial mediators of the association between hypertension and sleep duration [9–11]. It has been demonstrated that sleep restriction leads to an imbalance of appetite-regulating hormones that may result in increased food intake [6]. The increased hunger may be satisfied by unhealthy food choices that impair cardiovascular health, which could potentially increase arterial stiffness, an effect that is also caused by sleep problems themselves [12].

Mediation analyses are important to disentangle the magnitude of an exposure's effect that may be explained by another variable [13]. To obtain a better understanding of the effect of sleep duration and sleep difficulties on hypertension, it is important to assess what mediator variables may exist in this pathway and how much of the direct effect they explain.

In many developing countries, like Peru, the prevalence of hypertension differs within the country, highlighting the need to perform epidemiological studies in a variety of settings [14, 15]. Studies on the association between sleep duration or difficulties and hypertension have been conducted mostly in developed countries, with the exception of Brazil [3, 9, 16]. Complementing the available evidence with results from resource-constrained settings in developing countries – where risk factors, exposures, and health outcomes have different distributions compared with developed countries – is much needed. While it has been reported that Peruvians have adequate sleep duration [17], there is a lack of information on the association between sleep patterns and hypertension, or the possible role of BMI in any observed association. Consequently, we aimed to estimate the association between sleep duration and sleep difficulties with hypertension, and to assess and quantify any mediation effect of BMI, on more than 2300 individuals in Peru.

## Methods

### Study design and data source

This is a secondary analysis of the baseline assessment of an implementation study in Peru. Further details about the implementation study have been published elsewhere [18]. Briefly, the study was conducted in six villages in Tumbes, a region in Northern Peru and at sea level. Tumbes is a semi-urban setting where areas traditionally used for agricultural and fishing are undergoing urbanization [18]. Current statistics (2013) indicate a total population of 237 685 people, and the 3.5% of them have no formal education. In 2012, 61.2% of the population had health insurance. As much as 14.4% of the population was considered poor and 1.4% extremely poor.

### Participants

Eligible subjects were identified from the most updated census of six randomly selected villages, out of 20 potential villages in which there were between 300 and 600 inhabitants. Participants were 18+ years old, full-time residents in the area, and capable of understanding procedures and giving informed consent. All houses were approached, and all eligible members of a household were included. Exclusion criteria included having any mental illness preventing the participant from providing informed consent, as well as self-reported diagnosis of chronic kidney disease or heart disease, as required in the original implementation study [18]. The sample was selected to participate in an implementation study, and it is not representative of the whole area or Tumbes.

### Variables

Further details on data collection procedures have been published elsewhere [18]. Briefly, clinical evaluations were performed by trained health personnel and included blood pressure, height and weight measurements in each household. Blood pressure was measured three times after a 5-min resting period, and the mean of the last two measurements was used for the analysis. Participants were not fasting or off medication. Standard and calibrated devices (OMRON HEM-780, Tokyo, Japan) were used throughout the data collection process. All other variables were assessed with standardized questionnaires. All the study subjects answered the same questionnaires, which included domains from the WHO STEPS approach questionnaire (questions about alcohol consumption and smoking) and the short version of the IPAQ (physical activity questions).

#### Outcome variable

The outcome variable was a binary variable that indicated if hypertension was present (yes/no), defined as one or more of the following: (a) systolic blood pressure ≥140 mmHg, (b) diastolic blood pressure ≥90 mmHg, (c) self-report of physician diagnosis, or (d) current use of antihypertensive medication [19].

#### Exposure variables

There were two exposure variables of interest: short sleep duration and sleep difficulties. Each exposure of interest was subject of a separate mediation analysis. Sleep duration was assessed with the question: *On average, in the last year, how many hours did you sleep in an average day (including naps)?* Sleep duration was defined using the cut-off points as per National Sleep Foundation recommendations [20]: the reference was sleeping between 7 and 9 h for adults, and between 7 and 8 h for older adults (aged 65+). Individuals who obtained less or more sleep duration were categorized as having short or long sleep duration, respectively.

Based on similar definitions in previous studies [21–23], sleep difficulties were defined with a combination of two questions: *During the last month, have you had difficulty falling asleep?* and *During the last month, how frequently have you woken up during the night?* Possible answers to each question were: *almost never*, *sometimes*, and *frequently*. Participants who answered *sometimes* or *frequently* to both questions were classified as having sleep difficulties; those who answered *almost never* to both questions were considered not to have sleep difficulties.

#### Other variables

Other variables included sex; age (18–29, 30–44, 45–60, 60+); assets index (in tertiles) based on assets and household facilities; physical activity assessed with the IPAQ (International Physical Activity Questionnaire) and classified as low, moderate, or high [24]; heavy drinker, defined as having a hangover or ≥6 drinks on the same occasion at least once per month (yes/no); current smoker (yes/no); depression assessed with the PHQ-9 (Patient Health Questionanire-9) score and classified as ≥15 or <15 because this cut-off has the highest specificity [25]; obesity as BMI ≥30 kg/m^2^; and village in which the participant lived. BMI (kg/m^2^) as a numerical continuous variable was assessed as a mediator variable. Sex was assessed in interaction analyses to explore whether the associations of interest varied among men and women.

### Statistical methods

All analyses were conducted using Stata version 13.0 (STATA Corp, College Station, Texas, USA). To describe numerical variables, we used means and standard deviations (s.d.), and to describe categorical variables, we used proportions with 95% confidence intervals (95% CIs). To compare numerical variables, we used the two-sample *t* test, and to compare categorical variables, we used the χ^2^ test.

**Figure 1:**
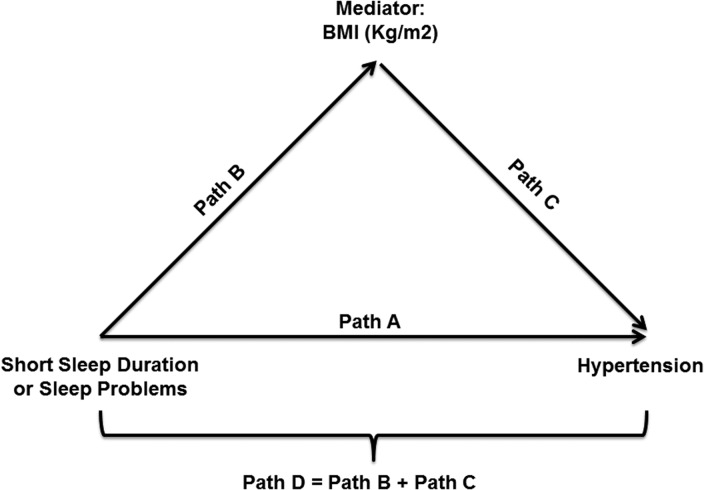
Diagram for the mediation analysis. Baron and Kenny defined a set of models that must be met to define a mediator variable [26]: (i) there is a significant association between the exposure and the outcome (path A); (ii) there is a significant association between the exposure and the mediator (path B); (iii) including the exposure and mediator in the model, there is a reduction of the association estimate between the exposure and outcome (estimates in path D < path A). These requisites for paths A, B, and D should be met to consider a variable to be a mediator.

Prevalence ratios (PR) and 95% CI were calculated using generalized linear models with robust variance to account for the cluster effect due to more than one member per household. The PR has a similar interpretation as odds ratio, but they give more conservative results in cross-sectional studies [26]. Regression models were constructed as depicted in Fig. 1. In addition, an adjusted model was fitted including all other variables apart from BMI. The adjusted model, including obesity, was fitted to assess if there was association between sleep duration or difficulties and hypertension, independent of potential confounders.

Because previous studies have reported different results in the association between sleep indicators and hypertension for men and women [3, 5], we assessed if the regression model including a multiplicative interaction between sex and the sleep indicator was better than a mode with either one independently. The after estimation likelihood-ratio test was used. However, regardless of the interaction results, regression models were stratified by sex for comparison purposes. In these stratified models, sex was not included as a co-variable.

In order to determine if BMI was a mediator of the association between the sleep duration or sleep difficulties and hypertension, we first explored mediation with a set of equations (Fig. 1) [26]. A mediator variable stands in the causal path between an exposure and an outcome. In addition, the mediator variable affects the outcome and is itself affected by the exposure [27]. Mediation analyses were conducted for the two exposure variables, and separately by sex. To quantify the mediation effect, we used the *ldecomp* command in STATA, which decomposes the total effect into direct and indirect effects [28].

### Ethics

Ethical approval was obtained from two Institutional Review Boards: Universidad Peruana Cayetano Heredia in Peru and Johns Hopkins University in the USA. The original implementation study was registered in ClinicalTrials.gov (NCT01960972). The authors assert that all procedures contributing to this work comply with the ethical standards of the relevant national and institutional committees on human experimentation and with the Helsinki Declaration of 1975, as revised in 2008.

## Results

### Participants

At baseline, a total of 2376 individuals were evaluated, and the 1.6% was excluded due to missing data, leaving full data available for 2338 participants. The participant mean age was 43.3 (s.d. 17.2) years, and 50.7% of the study population were women. On average, participants slept 7.8 (s.d.: 1.3) h, and 24.4% (95% CI 22.7–26.2%) reported having sleep difficulties (Table 1). The overall prevalence of hypertension was 18.2% (95% CI 16.7–19.8%). Table 2 shows participants’ characteristics by hypertension status.

**Table 1.** Self-reported sleep duration and sleep difficulties

Sleep patterns	% (95% CI)		
	Overall	Women	Men
Sleep duration^a^	*N* = 2338	*N* = 1185	*N* = 1153
7–9 h	73.1 (71.2–74.8)	76.9 (74.4–79.1)	69.1 (66.4–71.7)
<7 h	15.3 (13.9–16.8)	10.5 (8.8–12.3)	20.2 (18.0–22.6)
>9 h	11.7 (10.4–13.0)	12.7 (10.9–14.7)	10.7 (9.0–12.6)
Sleep difficulties	*N* = 2303	*N* = 1169	*N* = 1134
No	75.6 (73.8–77.3)	70.7 (68.1–73.3)	80.6 (78.2–82.8)
Yes	24.4 (22.7–26.2)	29.3 (26.7–31.9)	19.4 (17.2–21.8)
Difficulties getting to sleep	*N* = 2338	*N* = 1185	*N* = 1153
Almost never	75.7 (73.9–77.4)	71.0 (68.3–73.5)	80.6 (78.2–82.8)
Sometimes	20.4 (18.8–22.1)	24.1 (21.7–26.6)	16.7 (14.6–18.9)
Frequently	3.9 (3.2–4.8)	5.0 (3.9–6.4)	2.8 (2.0–3.9)
Waking up at night	*N* = 2338	*N* = 1185	*N* = 1153
Almost never	74.7 (72.9–76.4)	70.0 (67.3–72.5)	79.6 (77.2–81.9)
Sometimes	21.3 (19.7–23.0)	24.9 (22.5–27.4)	17.6 (15.5–19.9)
Frequently	4.0 (3.3–4.9)	5.2 (4.0–6.6)	2.8 (2.0–3.9)

a Recommended sleep duration defined as 7–9 h for adults aged 65 years and under, and 7–8 h for adults aged 65+ years old.

**Table 2.** Characteristics of the study population according to hypertension status

Variable	Hypertension		*p*^a^
	No	Yes	
Age (years)	*n* = 1912	*n* = 426	
18–29	31.4	4.7	<0.001
30–44	36.4	16.7	
45–59	20.2	30.5	
60+	12.0	48.1	
Mean age (years)	39.9 (±15.3)	59.0 (±16.8)	<0.001
Sex	*n* = 1912	*n* = 426	
Female	49.1	57.8	0.001
Male	50.9	42.3	
Assets index	*n* = 1876	*n* = 419	
Bottom	28.8	32.0	0.238
Middle	34.3	30.3	
Top	36.8	37.7	
Physical activity	*n* = 1912	*n* = 426	
Low	65.1	73.2	<0.001
Moderate	25.0	22.1	
High	10.0	4.7	
Heavy drinker	*n* = 1912	*n* = 426	
No	93.0	95.8	0.038
Yes	7.0	4.2	
Current smoker	*n* = 1902	*n* = 425	
No	88.2	91.8	0.036
Yes	11.8	8.2	
Depression	*n* = 1912	*n* = 426	
No	98.2	97.7	0.434
Yes	1.8	2.4	
BMI (kg/m^2^)	26.9 (± 4.5)	28.7 (±4.7)	<0.001
Obesity	*n* = 1868	*n* = 415	
No	77.6	62.9	<0.001
Yes	22.4	37.1	
Sleep (hours)	7.8 (± 1.3)	7.7 (±1.4)	0.061
Sleep duration	*n* = 1912	*n* = 426	
Suggested^b^	75.0	64.6	<0.001
Less than suggested	14.2	20.2	
More than suggested	10.9	15.3	
Sleep difficulties	*n* = 1882	*n* = 421	
No	79.3	58.9	<0.001
Yes	20.7	41.1	

a χ^2^ (categorical variables) or two-sample *t* test (continuous variables).

b Recommended sleep duration defined as 7–9 h for adults aged 65 years and under, and 7–8 h for adults aged 65+ years old. Percentages are presented for categorical variables, while for numerical variables the mean (±s.d.) is depicted.

When sleep duration was the exposure of interest, a model including its interaction with sex was not better than the simple regression model (*p* = 0.97); likewise, when the model used sleep difficulties as the exposure, including the interaction term did not improve it (*p* = 0.24).

### Short sleep duration and hypertension

Relative to subjects with hypertension, we found that individuals reporting the recommended sleep duration were more likely to be normotensive (*p* < 0.001, Table 2). In the adjusted model, including men and women, there was no association between short sleep duration and hypertension: PR = 1.21 (95% CI 0.99–1.49, Table 3).

**Table 3.** Association between short sleep duration or sleep difficulties and hypertension

	PR (95% CI)				
	Path A	Path B	Path C	Path D	Adjusted model
Overall					
Sleep duration (reference: suggested sleep duration)	*n* = 2066	*n* = 2044	*n* = 2316	*n* = 2044	*n* = 1976
Less than suggested	**1.48** (**1.19–1.84)**	1.44 (0.86–2.42)	**1.07** (**1.05–1.09)**	**1.47** (**1.18–1.82)**	1.21 (0.99–1.49)
Sleep difficulties (reference: no)	*n* = 2306	*n* = 2281	*n* = 2316	*n* = 2281	*n* = 2202
Yes	**2.14 (1.81–2.53)**	1.32 (0.84–2.07)	**1.07 (1.05–1.09)**	**2.09 (1.77–2.47)**	**1.43 (1.22–1.68)**
Women					
Sleep duration (reference: suggested sleep duration)	*n* = 1036	*n* = 1020	*n* = 1167	*n* = 1020	*n* = 980
Less than suggested	**1.60 (1.18–2.17)**	1.05 (0.41–2.64)	**1.05 (1.03–1.07)**	**1.62 (1.19–2.19)**	1.11 (0.82–1.51)
Sleep difficulties (reference: no)	*n* = 1170	*n* = 1151	*n* = 1167	*n* = 1151	*n* = 1106
Yes	**2.30 (1.85–2.87)**	1.24 (0.67–2.29)	**1.05 (1.03–1.07)**	**2.31 (1.86–2.88)**	**1.68 (1.36–2.07)**
Men					
Sleep duration (reference: suggested sleep duration)	*n* = 1030	*n* = 1024	*n* = 1149	*n* = 1024	*n* = 996
Less than suggested	**1.55 (1.15–2.10)**	**2.88 (1.61–5.17)**	**1.09 (1.06–1.12)**	**1.43 (1.06–1.95)**	1.30 (0.98–1.72)
Sleep difficulties (reference: no)	*n* = 1136	*n* = 1130	*n* = 1149	*n* = 1130	*n* = 1096
Yes	**1.90 (1.43–2.52)**	0.91 (0.47–1.73)	**1.09 (1.06–1.12)**	**1.83 (1.37–2.43)**	1.12 (0.86–1.47)

Path A, the exposure is sleep duration or sleep difficulties and the outcome hypertension. Path B, the exposure is sleep duration or sleep difficulties and the outcome is BMI. Path C, the exposure is BMI and the outcome is hypertension. Path D, the exposure is sleep duration or sleep difficulties and the outcome is hypertension, adjusted for BMI. Adjusted model included obesity status, sex, age in categories, assets index in tertiles, physical activity, heavy drinker, current smoker, and depression. All models adjusted by the village in which the participant live. In bold are presented the statistically significant results (*p*<0.05).

### Sleep difficulties and hypertension

We found that sleep difficulties were strongly associated with hypertension (*p* < 0.001, Table 2). In the multivariable model when compared with individuals without sleep difficulties, and including both men and women, there was 43% higher prevalence of hypertension among those with sleep difficulties (Table 3). When the results were stratified by sex, this association only remained among women with 68% higher prevalence (Table 3).

### Mediation analysis

In combined analyses of men and women, and according to our definition, BMI was not a mediator of either the associations of interest. In sex-specific analyses, we found that BMI partly mediated the association between short sleep duration and hypertension in men only (Table 3): among men, BMI accounted for 18.6% of the effect of short sleep duration on hypertension.

## Discussion

### Main results

In our sample of 2338 Peruvian men and women living in a resource-limited setting, we found evidence of an increased prevalence of hypertension among those with sleep difficulties (difficulties to fall asleep and waking-up at night), but not with short sleep duration (<7 h). The results also suggested that either association of interest did not change according to sex. In the overall model, BMI could not be classified as a mediator variable; however, BMI seems to be a partial mediator of the association between sleep duration and hypertension in men only. Because the results are not conclusive to draw strong recommendations, they should be verified by future studies aiming to assess this, and other potential mediators, following more comprehensive methods in cross-sectional and prospective studies.

### Comparison with previous studies

There is evidence of an association between short sleep duration and hypertension from both prospective [3, 4, 29], and cross-sectional studies [3, 5]. However, several cross-sectional [3, 5] studies reported no association while others found the association is sex-dependent or only in a particular age range [3, 5, 9]. Of particular interest, a cross-sectional study in the USA with a Hispanic population reported that short sleep duration was not associated with hypertension [30]. Dissimilar results may be explained by greater mean age in some studies [3, 5, 21], different distribution of the variables of interest (i.e. hypertension, short sleep duration) [17], and differences in environmental factors, such as pollution or noise [31]. Further research should be conducted on the role of environmental factors in the association between sleep (duration or problems) and hypertension in developing settings undergoing urbanization.

Some prospective studies have reported that sleep difficulties are associated with a higher risk of hypertension [4], and yet conclusive results were not retrieved by other cross-sectional studies using our same definition of sleep difficulties [21–23]. However, some of the studies with a prospective design included a third component in their sleep difficulties definition: waking up too early in the morning. We did not include this component, which could explain the different results. This may imply that each component has a different effect on the association estimates. In fact, prospective studies have reported different risk estimates for hypertension using each of the three components individually as well as in different combinations [32, 33]. Overall, we found similar association estimates for each component of the sleep difficulties definition. Thus, the third component could have radically changed the estimates. This warrants verification in a similar population to that one in this study.

### Interpretation of results

Overall, we found that hypertension was associated with sleep difficulties, but not with short sleep duration. This could indicate that these risk factors may be linked to hypertension through different pathways [4]. In the overall model, BMI did not prove to be mediator variable, thus other potential mediators need to be explored. However, our results suggest that BMI partially mediates the association between sleep duration and hypertension in men only; this could suggest that the causal pathways between the two exposures of interest and hypertension may be different for men and women. This is relevant as there is still room to study sleep difficulties, or symptoms of other sleep disorders (e.g. restless legs syndrome, sleep apnea, periodic limb movement disorder, etc.), in terms of the physiological pathways that lead to hypertension [34, 35]. For example, insomnia leads to a hyper-arousal state in which the sympathetic nervous system activity is increased; when the central nervous system is chronically activated, subjects are at higher risk for cardiovascular events [34]. Unlike short sleep duration, sleep disorders require the development of interventions beyond the simple recommendation to sleep longer or at least what is suggested for the age range [20]. Consequently, further studies should be conducted to confirm the mediation effect of BMI according to sex. This would help to display gender-specific interventions for each exposure herein assessed.

Conducting a mediation analysis gives evidence on what variables account for the effect of a given exposure on an outcome of interest (indirect effect). Our results do not support the hypothesis that BMI is a mediator in the associations of interest including both men and women. Nevertheless, and although preliminarily, our results suggest that BMI could be a mediator in men only; this observation warrants careful interpretation as well as further verification.

The role of BMI in the association between sleep (duration and problems) with hypertension should not be negligible; nevertheless, further studies must be conducted to verify our results before strong recommendations are made for subjects in developing countries. Furthermore, there is a need to study what other mediator variables are there in the associations of interest, and which ones have the greatest indirect effect, so can be subject of primary prevention strategies.

This work included a population undergoing epidemiological transition and urbanization. Similar populations have not been much included in epidemiological studies linking sleep and health outcomes. Therefore, our results underscore the importance of improving sleep health in resource-limited settings to minimize their negative health effects. This could be achieved by directly addressing sleep health or by addressing the mediator that impair it.

### Strengths and limitations

The main strength of the study is the large sample size of a resource-limited setting, using standardized techniques for measuring height, weight, and blood pressure that potentially allow comparability with other studies.

This study also had several limitations. First, the cross-sectional design does not rule out reverse causation. Future studies should follow a prospective design to confirm the long-term effect of BMI as a partial mediator of the associations of interest. Moreover, the mediation effect of BMI in the association between sleep patterns and hypertension should be further explored considering environmental variables, such as noise and air pollution [31]. Second, information about sleep duration and difficulties was self-reported. Self-reported information could have resulted in recall bias, particularly as the questions for some variables required the participant to remember events from the last year (e.g. sleep duration). This limitation, shared with other studies, could be addressed in future studies by using other methods such as 24-h diaries [3, 4, 9]. The gold-standard method would be to conduct a polysomnography study, though this can be challenging in population-based studies with a large sample size in remote settings. Although the exposures of interest relied on self-reported data, it has been suggested that there is a moderate correlation between subjectively measured and objectively measured sleep duration [36]. On top of that, Lauderdale *et al*. found that subjects who sleep 5 h report sleeping 1.2 h more [36]. Thus, our results are conservative and draw attention as there may be a much higher prevalence and burden of short sleep duration. In addition, sleep patterns over the last five decades have a similar trajectory assessed with objective and subjective measures [37]. Consequently, in spite of the differences between these methods, they seem to give a similar overall picture. Third, this is a secondary analysis, and thus the sample and data were not exclusively collected to answer this research question. Consequently, some of the regression estimates could have been underpowered; likewise, there could have been lack of power to find a significant interaction effect of sex. Nevertheless, most of the estimates showed a significant association, in spite of a possible lack of power. This calls to confirm our results because the association and mediation effect could be stronger than reported herein. Fourth, regarding the questions to assess sleep quality, other studies used similar questions [21–23] and we do not believe these will operate differently in our study: the questions are fairly easy to understand and since they need the participant to recall the previous months, there should be low recall bias. Fifth, our results could have changed toward the null association due to the effect of unmeasured confounders, such as drug use, household crowding, children/toddlers in the household, or shift work status. This outlines the need to conduct more studies about sleep health in resource-limited settings, where these features are rather common. Some bias in mediation analysis has been described [13, 38]. The lack of further analyses to address these biases in mediation analysis could be a limitation of this study. However, we aimed to verify if BMI was a mediator of the association of interest and how much of the association it represents. Conducting a complete multiple-mediator model was beyond the scope of this paper.

## Conclusions

Among men and women, sleep difficulties, but not short sleep duration, were associated with a higher prevalence of hypertension; moreover, BMI did not seem to mediate these associations. This study highlights the need to conduct more studies relating to sleep health in resource-limited settings, and to further explore BMI and other variables as possible mediators in the association between sleep health and hypertension.
